# Correction: Adjustment Disorders as a Stress-Related Disorder: A Longitudinal Study of the Associations among Stress, Resources, and Mental Health

**DOI:** 10.1371/journal.pone.0101377

**Published:** 2014-06-27

**Authors:** The PLOS ONE Staff

## Notice of Republication

This article was republished on May 30, 2014, to correct the order of Figures 1-3. The publisher apologizes for the error. Please download this article again to view the correct version. The originally published, uncorrected article and the republished, corrected article are provided here for reference.

## Supporting Information

File S1 Originally published, uncorrected article.
(PDF)

File S2 Republished, corrected article.
(PDF)
