# Supplementary material for: Correction: Adjustment Disorders as a Stress-Related Disorder: A Longitudinal Study of the Associations among Stress, Resources, and Mental Health
Source: PLoS One. 2014 Jun 27;9(6):e101377. doi: 10.1371/journal.pone.0101377 (PMC4074194; doi:10.1371/journal.pone.0101377)
Supplement: File S1 — Originally published, uncorrected article. (PDF) [file pone.0101377.s001.pdf]

PLOS | ONE

Adjustment Disorders as a Stress-Related Disorder: A Longitudinal Study of the Associations among Stress, Resources, and Mental Health

Rüya-Daniela Kocalevent[1]*, Annett Mierke[2], Gerhard Danzer[2], Burghard F. Klapp[2]

1 Institute and Policlinic for Medical Psychology, University Medical Center, Hamburg-Eppendorf, Germany, **2** Department of Psychosomatic Medicine, Charité University Medicine, Berlin, Germany

Abstract

Objective: Adjustment disorders are re-conceptualized in the DSM-5 as a stress-related disorder; however, besides the impact of an identifiable stressor, the specification of a stress concept, remains unclear. This study is the first to examine an existing stress-model from the general population, in patients diagnosed with adjustment disorders, using a longitudinal design.

Methods: The study sample consisted of 108 patients consecutively admitted for adjustment disorders. Associations of stress perception, emotional distress, resources, and mental health were measured at three time points: the outpatients' presentation, admission for inpatient treatment, and discharge from the hospital. To evaluate a longitudinal stress model of ADs, we examined whether stress at admission predicted mental health at each of the three time points using multiple linear regressions and structural equation modeling. A series of repeated-measures one-way analyses of variance (rANOVAs) was performed to assess change over time.

Results: Significant within-participant changes from baseline were observed between hospital admission and discharge with regard to mental health, stress perception, and emotional distress ($p<0.001$). Stress perception explained nearly half of the total variance (44%) of mental health at baseline; the adjusted R^2 increased (0.48), taking emotional distress (i.e., depressive symptoms) into account. The best predictor of mental health at discharge was the level of emotional distress (i.e., anxiety level) at baseline ($\beta = -0.23$, $R^2_{corr} = 0.56$, $p<0.001$). With a CFI of 0.86 and an NFI of 0.86, the fit indices did not allow for acceptance of the stress-model ($C_{min}/df = 15.26$; RMSEA = 0.21).

Conclusions: Stress perception is an important predictor in adjustment disorders, and mental health-related treatment goals are dependent on and significantly impacted by stress perception and emotional distress.

Citation: Kocalevent R-D, Mierke A, Danzer G, Klapp BF (2014) Adjustment Disorders as a Stress-Related Disorder: A Longitudinal Study of the Associations among Stress, Resources, and Mental Health. PLoS ONE 9(5): e97303. doi:10.1371/journal.pone.0097303

Editor: Gabriel S. Dichter, UNC Chapel Hill, United States of America

Received October 30, 2013; **Accepted** April 17, 2014; **Published** May 13, 2014

Copyright: © 2014 Kocalevent et al. This is an open-access article distributed under the terms of the Creative Commons Attribution License, which permits unrestricted use, distribution, and reproduction in any medium, provided the original author and source are credited.

Funding: No current funding sources for this study.

Competing Interests: The authors have declared that no competing interests exist.

* E-mail: r.kocalevent@uke.de

Introduction

Adjustment disorders (ADs) have recently been defined as a stress response syndrome [1]. This new diagnostic concept was addressed in the preparation of the DSM-5 and the ICD-11. Stressful events or continuing unpleasant circumstances are the primary and overriding causal factors of ADs, and this disorder does not occur without such effects [2]. Specifically, "The disorders in this section can thus be regarded as maladaptive responses to severe or continued stress, in that they interfere with successful coping mechanisms and therefore lead to problems of social functioning" [2]. While the impact of stressors in this new conceptualization of ADs is well specified, there is a lack of empirical data examining the associations among stress perception, resources, and emotional distress on mental health in ADs. Maercker and colleagues [1] required new empirical data when preparing the DSM-5 and the ICD-11 diagnostic nomenclature for ADs as a stress-related disorder.

This unresolved specification of the stress construct in diagnosed ADs is also reflected in the high variance of prevalence rates. Maercker and colleagues [1] reported a 12-month AD prevalence of 0.9–2.3% in Germany, depending on whether functional impairments were taken into account. An estimated prevalence of 0.5% was derived using data from the European Outcome of Depression International Network study [3]. The prevalence of ADs in China is <1% [4]. A representative study of ADs among 65- to 96-year-olds from Switzerland yielded a prevalence of 2.3% [5]. In a cross-sectional study on primary care and psychiatric outpatients over the age of 60 with anxiety symptoms alone, ADs yielded a high prevalence of 3.7% [6]. The prevalence of ADs in primary care was 2.9% in a representative cross-sectional study [7]. ADs continue to be diagnosed in a clinical setting range, with up to 12% of referrals in consultation-liaison psychiatry [8].

Empirical data concerning the specification of the stress construct in ADs are scarce. Previous cross-sectional empirical

findings indicate higher self-perceived stress scores among patients with ADs compared with those with anxiety disorders or other mental disorders. Furthermore, the mental dimension of quality of life in people with ADs was higher than in those with other mental disorders but lower than in patients with somatic disorders, with women scoring lower than men [7,9].

To date, theoretical model assumptions of the processes within stress response syndromes are limited to post-traumatic stress disorders [10–12]. These so-called cognitive models of persisting stress seek to explain three forms of cognitive stress processing: a) successful completion, b) chronic cognitive processing, and c) the inhibition of cognitive processing. A recent study explored whether severe life-events (i.e., major stressors) were associated with ADs according to a postulated stress-response model of ADs [13,14]. The proposed model described ADs as particular forms of the (dis)stress response syndrome, in which intrusion, avoidance of reminders, and failure to adapt to the life-events are the central processes and symptoms [14].

According to a transactional model of stress, individuals who perceive stress examine present, available resources [15,16]. Lazarus and Folkman's hypothesis was confirmed in the general population as an accurate operationalization of the stress construct with cross-sectional empirical data to be used for further analysis [17,18]. These studies contributed to the transactional understanding of stress by identifying the direct effects of resources on the perception of stress and the indirect effects on health-related aspects [17,18].

The authors created a model assumption on the basis of the aforementioned previous results, in order to study these complex reciprocal stress relationships in the current study (Figure 1). The total effects of resources (T1) on mental health (T3) are postulated to be decomposed into the direct effect of resources (T1) on mental health (T3) and the indirect effect mediated via stress (T1).

The purpose of this longitudinal study was to examine an existing cross-sectional stress-model from the general population, in patients diagnosed with adjustment disorders. According to the stress-model, stress comprises of personal resources, stress perception, and mental health. At first, we assessed stress perception, resources, emotional distress, and the mental health dimension of quality of life, among patients clinically diagnosed with ADs. We hypothesized that stress, including stress perception and emotional distress, would predict mental health in people with ADs. Structural equation modeling was used to test for the stress-model (Figure 1) in ADs.

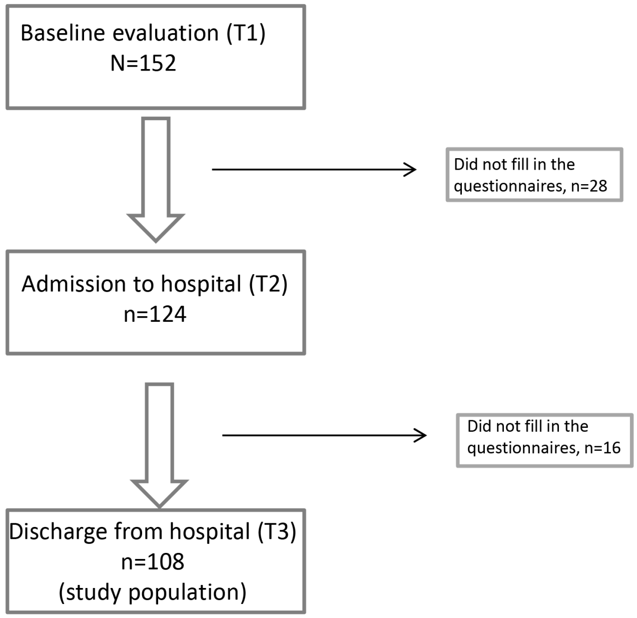

Figure 1. Postulated stress model.
doi:10.1371/journal.pone.0097303.g001

Methods

Ethics Statement

The institutional review board of Charité University Medicine Berlin approved this study. Written informed consent was obtained, and all clinical investigations were conducted according to the principles expressed in the Declaration of Helsinki.

Sample

The data from 108 consecutive patients who were diagnosed with ADs and treated at the psychosomatic inpatient department at Charité University Medicine Berlin, Germany between 01/2007 and 02/2013 were analyzed. All patients completed all questionnaires during an outpatient evaluation session (either at a visit to the outpatient department or via a psychosomatic liaison service) at baseline (T1), at admission to the inpatient department (T2), and at discharge (T3; see Figure 2). The patients included in this study waited between one day and 8 weeks after the initial visit for inpatient treatment. The treatment lasted 2–14 days.

Clinical Setting

The psychosomatic inpatient treatment included an extensive clinical interview by an experienced clinician, questionnaires that were displayed on personal digital assistants (PDAs), and at least one counseling session with a trained psychologist. The inpatient treatment covered general treatment modules (different group therapies and psychoeducation for all patients) and disorder-specific treatment modules centered on stress regulation (for patients with stress response syndromes) based on cognitive-behavioral therapy with a strong physiotherapeutic emphasis on single-person therapy, massage, sauna, swimming, and so on.

Instruments

In the study design, operationalization of different aspects of stress is used to specifically refer to resources, emotional distress, and the mental health dimension of quality of life among patients clinically diagnosed with ADs.

Resources

Brief resilient coping scale (BRCS). The BRCS is a 4-item questionnaire with a five-point rating scale that aims to identify individuals in need of resilient coping skill interventions [19]. The directions for the items are worded as follows: "Consider how well the following statements describe your behavior from 1 to 5, where 1 means the statement does not describe you at all and 5 means it describes you very well." The BRCS is considered to be sufficiently valid and reliable (Cronbach's $\alpha = .70$) [20].

Questionnaire on self-efficacy and optimism (SWOP). The SWOP evaluates self-efficacy and optimism. This instrument is considered valid and reliable (Cronbach's $\alpha = .79$) [21]. The questionnaire is composed of 9 items with four possible response categories: "not true," "most likely not true," "most likely true," and "true." Expectations of self-efficacy were defined as a source of generalized problem solving, which reflects an individual's sense of his or her own competency and ability. Optimism was defined as an individual's ability to channel an

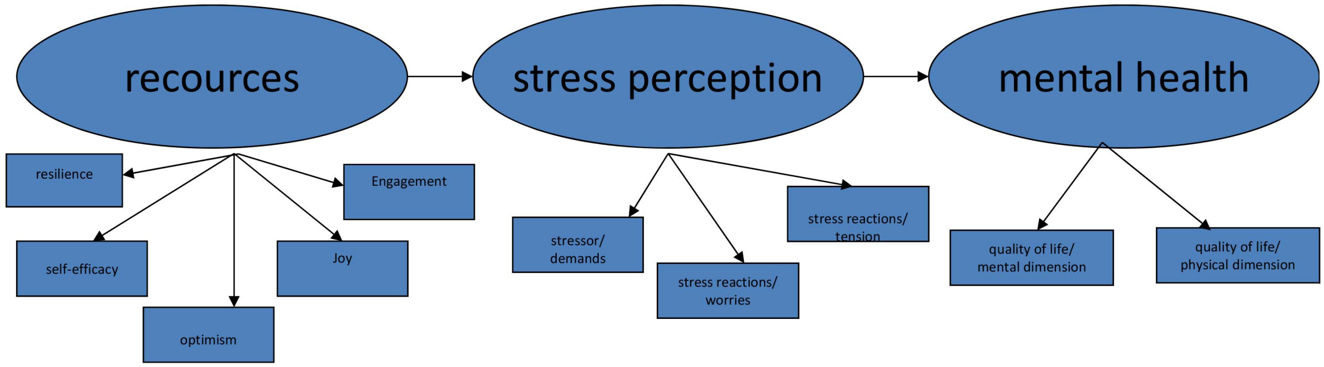

Figure 2. Flow-chart of the study participants.
doi:10.1371/journal.pone.0097303.g002

attitude in such a way that it will have an advantageous effect in dealing with change across various levels [22].

Motivation (BSF). The BSF is one of the six mood scales of the Berlin Mood Questionnaire, which was used for stress modeling only to represent one of the indicator variables for the latent variable resources [23]. This scale was added to account for the amount of motivation for treatment. Earlier studies of emotional adaptation and health-behavior change have identified motivation as a resource for goal achievement (see [24] for an overview on the concept).

This validated questionnaire is a shortened and rescaled form of the multidimensional mood questionnaire of Hecheltjen and Mertensdorf, which is itself a translation of the mood adjective list of Nowlis [25,26].

Emotional Distress

Emotional distress is one aspect of stress among people with ADs. In addition to the perception of stress, this construct was operationalized as the level of anxiety and depressive symptoms according to the Patient Reported Outcomes Measurement Information System of the NIH in the US (www.nihpromis.org).

Perceived stress questionnaire (PSQ-20). The PSQ-20 was administered to measure perceived stress during the previous 4 weeks [27]. The criteria used to construct the questionnaire were as follows: (a) stress was recorded as subjective perceived stress; (b) stress factors and subjective perceived stress should be unspecific but apply to a variety of real-life situations (e.g., "You feel under pressure from deadlines"); and (c) perceived stress should be recorded independently of the current stage in the coping process. The PSQ-20 includes 20 items that are assigned to four scales (demands, joy, worries, and tension). These items are answered using a four-point rating scale (1 = almost never, 2 = sometimes, 3 = often, and 4 = always) with regard to the last 4 weeks. The psychometric properties of the PSQ are well documented (for an overview, see [18]).

Depressive symptoms (PHQ-9). Depressive symptoms were assessed using the nine-item PHQ depression module (PHQ-9) [28]. Each of the nine items corresponds to one of the DSM-IV diagnostic criterion A symptoms for major depressive disorder [29]. Participants reported how often each depressive symptom bothered them over the last two weeks. The response options are "not at all," "several days," "more than half the time," and "nearly every day" and scored as 0, 1, 2, and 3, respectively. PHQ-9 scores range from 0 to 27; scores of $\geq$5, $\geq$10, and $\geq$15 represent mild, moderate, and severe levels of depressive symptoms, respectively [30]. The psychometric properties of the PHQ-9 are well documented (for an overview, see [31]).

Anxiety (GAD-7). The GAD-7, which identifies likely cases of generalized anxiety disorder and assesses symptom severity, has high reliability and validity among primary care patients [32–34]. The items of the GAD-7 describe the most prominent diagnostic features of the DSM-IV diagnostic criteria A, B, and C for generalized anxiety disorder [29]. Participants use the GAD-7 to indicate how often they have been bothered by each of the 7 core symptoms of generalized anxiety disorder over the last 2 weeks. The response options are "not at all," "several days," "more than half the time," and "nearly every day" and scored as 0, 1, 2, and 3, respectively. Therefore, GAD-7 scores range from 0 to 21, and scores of $\geq$5, $\geq$10, and $\geq$15 represent mild, moderate, and severe anxiety symptom levels, respectively.

Mental health (SF-8). The SF-8 is a generic questionnaire concerning perceived health-related quality of life; it assesses the overall subjective state of health of adults with different diseases in relation to physical, psychological, and social aspects. The SF-8 one-item scales (general health, physical functioning, role physical, bodily pain, vitality, social functioning, mental health, and role emotional) as well as physical and mental summary measures (PCS-8 and MCS-8, respectively) are scored using norm-based scoring methods [35,36]. By adding a constant (regression intercept), the aggregate PCS-8 and MCS-8 scores are standardized to have the same mean as the SF-36 [37]. The SF-36 was constructed to survey health status in the Medical Outcomes Study [38]. The SF-36 has been used to compare the health-related quality of life of patients with various diseases across different cultures. The 8-item version was specifically developed for population-based questionnaires, but it is also used for clinically relevant subgroups.

Data Analyses

A series of repeated measures one-way analyses of variance (rANOVAs) with least significant difference (LSD) post-hoc tests were performed with regard to mental and physical health, stress perception, and emotional distress (e.g., depression and anxiety symptoms) to assess changes for each variable separately over time. This approach was used to test for possible within-group effects as well as interactions between age and gender with regard to mental health, stress perception, or emotional distress.

Multiple linear regressions were performed to examine the relationships among resources (BRCS and SWOP), stress perception (PSQ), and emotional distress (PHQ-9 and GAD-7) as independent variables and mental health (SF-8) as the dependent variable. Specifically, (a) resources, stress perception, and emotional distress at T1 were used to assess associations with mental

health at T1; (b) resources, stress perception, and emotional distress at T3 were used to assess associations with mental health at T3; and (c) resources, stress perception, and emotional distress at T3 were used to predict mental health at T3. Structural equation modeling was applied to test an earlier empirical conceptualization of a stress model [17,18] among people with ADs; this model was based on previous cross-sectional population-based studies of stress perception. Structural equation modeling exceeds multiple regression analysis because it deals with a system of regression equations; it also represents hypotheses regarding the means, variances, and covariances of observed data in terms of a smaller number of "structural" parameters defined by the hypothesized underlying model [39]. We used the comparative fit index (CFI; acceptable, ≥0.95; good, ≥0.97) and the normed-fit index (NFI; acceptable, ≥0.95; good, ≥0.97) following Tanaka [40].

All data analyses were conducted using AMOS 20 and SPSS with an α level of 5%.

Results

Sample Characteristics

Sample characteristics are described in Table 1. The questionnaires were presented via PDAs to all consecutive patients at T1, T2, and T3. 152 patients were approached, and a total of 124 patients were enrolled at T1. The response rate at T2 was 81.6%, whereas 108 patients responded at all three time points. Forty-four patients were lost to follow-up, resulting in a 29% reduction from T1 to T3. At T1, 76.3% of the patients were not taking medications, whereas 14.4% were taking antidepressants, 1.0% were taking a tranquilizer, 1.0% were taking a barbiturate, and 0.5% were taking neuroleptics. Furthermore, 1.5% of the patients were taking multiple types of the aforementioned medications. The medication data were missing for 5.2% of the patients.

Internal Consistency

The internal consistency (Cronbach's α) statistics for the scales were as follows: (a) The BRCS had an α of 0.78; the SWOP self-efficacy scale had an α of 0.79; the PSQ had an α of 0.80; the GAD-7 anxiety scale had an α of 0.87; the PHQ-9 had an α of 0.73 for; and the SF-8 had an α of 0.81.

Changes in Resources, Emotional Distress, and Mental Health

We observed significant within-subjects changes from T1 through T3 with regard to mental health, stress perception, and emotional distress ($p<0.001$). The mean of mental health rose significantly over the three time points, and emotional distress (in terms of perceived stress, depressive symptoms, and anxiety symptoms) decreased. LSD post-hoc tests revealed that the score differences from T2 to T3 were significant for depressive symptoms ($p<0.001$) but not for stress perception, anxiety symptoms, or mental health. Differences from T1 to T2 and from T1 to T3 were significant for all scale scores (Figure 3). Significant time*gender or time*age interaction effects were not observed with regard to mental health, stress perception, or emotional distress.

The 1st stepwise multiple linear regression analysis (cross-sectional analysis at T1) indicated that the mental health summary score was significantly associated with stress ($\beta = -0.48$, $p<0.001$) and depressive symptoms ($\beta = -0.28$, $p<0.001$; see Table 2). The stress scale was the strongest predictor of mental health, when taken into account only in a first model, as it explained 44% of the total variance ($R^2 = 0.44$). The Durbin-Watson statistic was between the critical values of $1.5< d <2.5$ ($d = 2.254$); therefore, we can assume that first-order linear auto-correlations were not present in our multiple linear regression data (i.e., no serial correlation among the residuals).

The 2nd stepwise multiple linear regression analysis (cross-sectional analysis at T3) produced Model 2, in which the association between mental health and perceived stress was again the strongest ($\beta = -0.35$, $p<0.001$) and significantly associated with depressive symptoms ($\beta = -0.23$, $p<0.001$) and anxiety symptoms ($\beta = -0.27$, $p<0.001$) (Durbin-Watson $d = 1.902$).

The 3rd stepwise multiple linear regression analysis (i.e., the longitudinal analysis from T1 to T3, see Table 2) yielded Model 3. The mental health summary score at T3 was only significantly associated with the anxiety level at T1 ($\beta = -0.53$, $p<0.001$) and explained 29% of the total variance ($R^2 = 0.29$; Durbin-Watson $d = 1.907$). Collinearity statistics (tolerance and variance inflation factor) indicated that multicollinearity was not an issue in these three stepwise multiple linear regressions. Thus, each independent variable uniquely predicted the dependent variable.

Proposed Stress Model of ADs

Finally, we investigated the relationship between mental health at T3 and resources and stress perception at T1 using a structural equation model of stress (Figure 1). This model was constructed in light of our previous cross-sectional population-based studies [17,18,41]. The latent variable, resources, was operationalized using the constructs "self-efficacy," "optimism," "joy," "resilience," and "motivation." Stress perception was operationalized as a latent variable based on a combination of the constructs "stressor" (demands) and "stress reactions" (tension and worries). With a CFI of 0.86 and an NFI of 0.86, the fit indices did not allow for acceptance of the stress-model ($C_{min}/df = 15.26$; $RMSEA = 0.21$).

Discussion

The role of stress among people diagnosed with ADs is often discussed, yet it remains under-researched [8]. Our study examined the longitudinal associations of stress perception, resources, emotional distress, and subjective mental health state among patients diagnosed with ADs to clarify the role of stress within this disorder.

Our selection of variables under investigation was derived from the ICD-11 Beta Draft, in which ADs are categorized under the heading of "Disorders Specifically Associated with Stress" [2]. The results of the present study revealed significant within-participant changes from T1 through T3 with regard to mental health, stress perception, and emotional distress ($p<0.001$). These findings correspond to other studies in which the primary aim was assessing the treatment effectiveness among people with ADs. Compared with untreated control groups, an intervention psychotherapy group of patients with ADs significantly improved with regard to symptoms and life satisfaction, and stable effects were observed at 3-month and 2-year follow-up evaluations [42,43]. Another follow-up study found that chronicity and behavioral symptoms were the strongest predictors of poor outcomes among psychiatric inpatients with ADs [44]. Readmission rates among people with ADs are significantly lower than those with other mental disorders, which might be due to the recovery effects of chronic stress; these effects are not valid for depressive syndromes [45]. Long-term work absences due to ADs are best predicted by comorbidity, followed by under employment with an age between 35 and 44 years [46]. This age range is typical for stress-related syndromes such as fatigue and high perceived stress in general [41]. The present study did not reveal significant time*gender or time*age

Table 1. Characteristics of the sample under investigation.

**Sample characteristics**	
n	108
Sex (men)	35%
Age, years	45.2 (range: 22–71)
Job status	
Employed	74.2%
Education	
None	1.3%
High School	52.9%
College	45.8%
Cohabitation or married	
Yes	63.2%
No	36.8%
Visited a physician before admission	84.5%

doi:10.1371/journal.pone.0097303.t001

interaction effects with regard to mental health, overall stress, or depression. Data from a recent representative population study suggested that the relationship between health and stress depends more on age than on gender (Kocalevent et al., unpublished data). The peak in the range between 16–40 years (i.e., early adulthood) might be due to the work- and family-related challenges during this period of life [47]. Recent studies of different health care settings investigating gender differences in stress processing can be divided into those which have shown that women tend to report more psychological stressors than men [48–53] and those that have reported no differences (or differences with small effect sizes) [18,54–58]. The population-based samples between the 1960s and 1980s [50] reported clearer gender differences and support the assumption that role differences have decreased over the past decades (at least in industrial countries), thereby accounting for the fading gender effects with regard to stress. However, differences in self-rated health status and quality of life remain disadvantageous for women, who are more likely to have less favorable socioeconomic statuses [35,59].

We found that stress was strongly correlated with mental health and explained nearly half of the variance in this variable at T1. Stress was also the strongest cross-sectional predictor of mental health at T3. The longitudinal effects of stress and resources on mental health among patients with ADs could not be established.

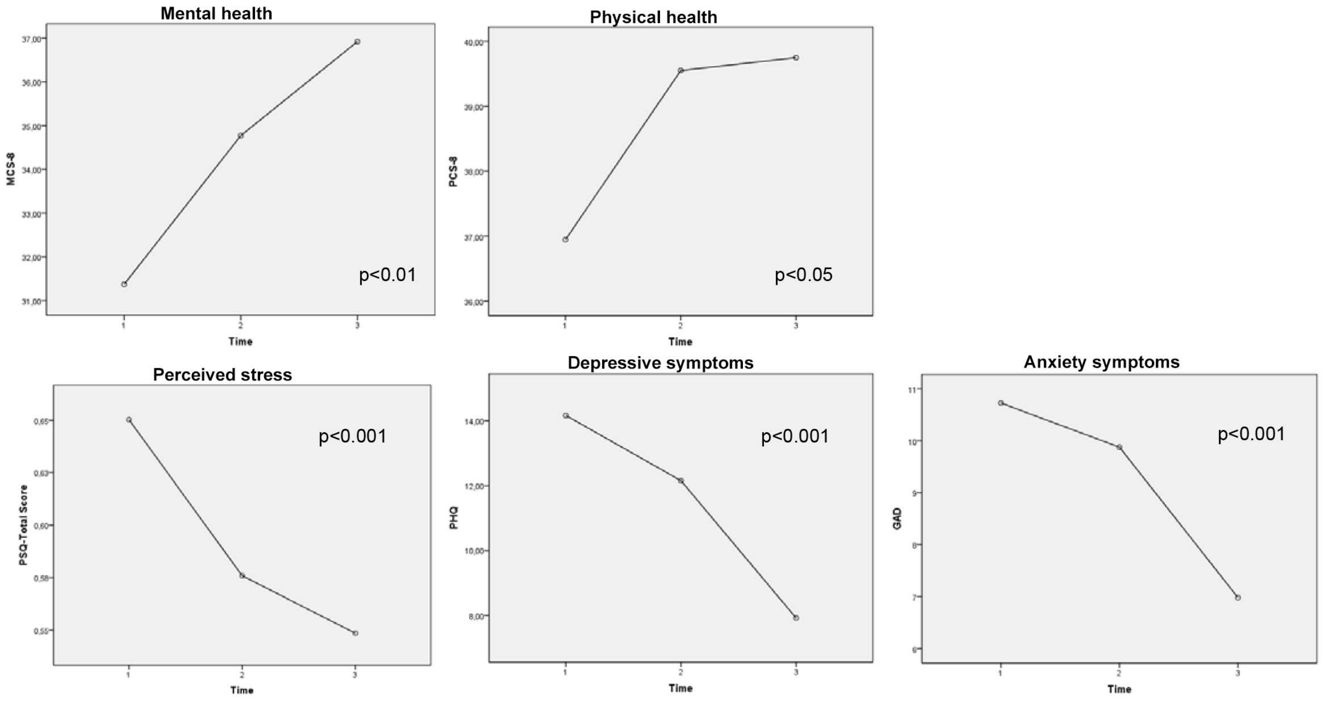

Figure 3. Results of the within-subjects effects. P-values are reported for significant changes from T1 through T3.
doi:10.1371/journal.pone.0097303.g003

Table 2. The results of stepwise multiple linear regressions with mental health as the dependent variable.

Measures of association			
**Independent Variables**	**Dependent Variable**	**β Coefficient***	**Adjusted R^2**
**First stepwise multiple linear regression**			
**T1 (baseline)**	**T1 (baseline)**		
**Model 1** PSQ (Perceived Stress)	SF-8 (Mental Health)	−**0.48**	**0.48**
PHQ-9 (Depressive Symptoms)		−**0.28**	
GAD-7 (Anxiety Level)		−0.18	
BRCS (Resource)		−0.07 n.s.	
SWOP (Resource)		−0.09 n.s.	
**Second stepwise multiple linear regression**			
**T3 (discharge)**	**T3 (discharge)**		
**Model 2** PHQ-9 (Depressive Symptoms)	SF-8 (Mental Health)	−**0.23**	**0.56**
PSQ (Perceived Stress)		−**0.35**	
GAD-7 (Anxiety Level)		−**0.27**	
BRCS (Resource)		–	
SWOP (Resource)		–	
**Third stepwise multiple linear regression**			
**T1 (baseline)**	**T3 (discharge)**		
**Model 3** GAD-7 (Anxiety Level)	SF-8 (Mental Health)	−**0.53**	**0.29**
PHQ-9 (Depressive Symptoms)		−0.18 n.s.	
PSQ (Perceived Stress)		−0.12 n.s.	
BRCS (Resource)		−0.06 n.s.	
SWOP (Resource)		−0.07 n.s.	

*Multiple linear regressions were performed ($p < 0.001$).
doi:10.1371/journal.pone.0097303.t002

Resources did not significantly predict mental health among people with ADs.

Our stress model hypothesis based on previous cross-sectional studies of the general population was not an accurate operationalization of the stress process among people with ADs according to the longitudinal data. However, the fit indices suggested that our results were in the right direction and need additional analysis with larger sample sizes. According to Tanaka [40], the ratio of the sample size to the number of free parameters should be 20:1; thus, our required sample size was N = 600, which was not accomplished within the current study timeline. Sufficient neurobiological models of prolonged (dis)stress syndromes/ADs are currently missing. A recent study protocol aimed to investigate the pharmacological interventions among people with ADs underpinned by biological parameters by assuming that the pathophysiology of this disorder is the same as that of major depressive disorder and generalized anxiety disorder [60].

Emotional distress (i.e., anxiety symptoms) at T1 best predicted the mental health of patients with ADs at T3. This change of symptoms might be due to the level of activation, dysregulation, or both of the HPA axis with regard to emotional distress, but would lead to an over-interpretation of the data in the current study [61]. In general, timing is an especially critical element because hormonal and neurotransmitter (e.g., dopamine) activity are elevated at the onset of a stressor; however, they decrease as time passes. Nevertheless, distress can also be characterized by the reduced tonic activity of the HPA axis, which might be related to chronicity, exposure to a stressor, and increased levels of harm avoidance, a typical behavior of anxiety-related symptoms and a postulated central process of AD according to Maercker and colleagues [14,62].

The recognition of ADs by primary care remains low [7]. A general practitioner identified only 2 of 110 cases using the SCID-I, yet 37% had at least one psychotropic prescription, which indicates the need for treatment [7]. The insufficiently elaborated assessment of ADs with regard to structured and standardized procedures (e.g., the CIDI) has recently been described and led to the development of a CIDI for patients with cancer (i.e., the CIDI-O) by adding the items for the diagnostic group of stress-related mental disorders [63].

The major strength of the study is its longitudinal design, which evaluated the temporal precedence and causality of the observed stress associations among people with ADs. Considering the longitudinal design, variability in patient treatment experience would likely be highly influential. Yet, results of a large routine practice sample showed that clients' mean pretreatment-posttreatment change was approximately constant regardless of treatment duration (in the range of 0 to 20 sessions) [64].

One limitation of this study was that all measures relied on self-reports without objective markers (e.g., biomarkers). Aside from this limitation, our theoretical conceptualization of ADs using a dysfunctional transactional stress model is relatively new. Although the model itself has been validated in several cross-sectional studies, it was only moderately associated with the present sample given its size. Furthermore, the variation in length hospital stay from T1 to T3 remains under-investigated in the proposed stress model. Despite these limitations, this longitudinal study represents

an important step in research on ADs in terms of its role as a stress response syndrome.

Author Contributions

Conceived and designed the experiments: RDK AM GD BFK. Performed the experiments: RDK BFK. Analyzed the data: RDK. Contributed reagents/materials/analysis tools: AM. Wrote the paper: RDK BFK.

References

1. Maercker A, Forstmeier S, Pielmaier L, Spangenberg L, Brahler E, et al. (2012) Adjustment disorders: prevalence in a representative nationwide survey in Germany. Soc Psychiatry Psychiatr Epidemiol 47: 1745–1752.
2. World Health Organization (2013) ICD-11 beta draft.
3. Ayuso-Mateos JL, Vazquez-Barquero JL, Dowrick C, Lehtinen V, Dalgard OS, et al. (2001) Depressive disorders in Europe: prevalence figures from the ODIN study. Br J Psychiatry 179: 308–316.
4. Phillips MR, Zhang J, Shi Q, Song Z, Ding Z, et al. (2009) Prevalence, treatment, and associated disability of mental disorders in four provinces in China during 2001–05: an epidemiological survey. Lancet 373: 2041–2053.
5. Maercker A, Forstmeier S, Enzler A, Krusi G, Horler E, et al. (2008) Adjustment disorders, posttraumatic stress disorder, and depressive disorders in old age: findings from a community survey. Compr Psychiatry 49: 113–120.
6. Arbus C, Hergueta T, Duburcq A, Saleh A, Le Guern ME, et al. (2013) Adjustment disorder with anxiety in old age: Comparing prevalence and clinical management in primary care and mental health care. Eur Psychiatry.
7. Fernandez A, Mendive JM, Salvador-Carulla L, Rubio-Valera M, Luciano JV, et al. (2012) Adjustment disorders in primary care: prevalence, recognition and use of services. Br J Psychiatry 201: 137–142.
8. Casey P, Bailey S (2011) Adjustment disorders: the state of the art. World Psychiatry 10: 11–18.
9. Jones R, Yates WR, Williams S, Zhou M, Hardman L (1999) Outcome for adjustment disorder with depressed mood: comparison with other mood disorders. J Affect Disord 55: 55–61.
10. Brewin CR, Dalgleish T, Joseph S (1996) A dual representation theory of posttraumatic stress disorder. Psychol Rev 103: 670–686.
11. Creamer M, Burgess P, Pattison P (1992) Reaction to trauma: a cognitive processing model. J Abnorm Psychol 101: 452–459.
12. Ehlers A, Clark DM (2000) A cognitive model of posttraumatic stress disorder. Behav Res Ther 38: 319–345.
13. Dobricki M, Komproe IH, de Jong JT, Maercker A (2010) Adjustment disorders after severe life-events in four postconflict settings. Soc Psychiatry Psychiatr Epidemiol 45: 39–46.
14. Maercker A, Einsle F, Kollner V (2007) Adjustment disorders as stress response syndromes: A new diagnostic concept and its exploration in a medical sample. Psychopathology 40: 135–146.
15. Lazarus R, Folkman S (1984) Stress, appraisal, and coping. New York: Springer Publishing Company.
16. Lazarus RS (2000) Toward better research on stress and coping. Am Psychol 55: 665–673.
17. Kocalevent RD, Klapp BF, Albani C, Brahler E (2013) [Associations of resources factors, chronic activated distress, and fatigue in the German general population]. Psychother Psychosom Med Psychol 63: 115–121.
18. Kocalevent RD, Levenstein S, Fliege H, Schmid G, Hinz A, et al. (2007) Contribution to the construct validity of the Perceived Stress Questionnaire from a population-based survey. J Psychosom Res 63: 71–81.
19. Sinclair VG, Wallston KA (2004) The development and psychometric evaluation of the Brief Resilient Coping Scale. Assessment 11: 94–101.
20. Ahern NR, Kiehl EM, Sole ML, Byers J (2006) A review of instruments measuring resilience. Issues Compr Pediatr Nurs 29: 103–125.
21. Scholler G, Fliege H, Klapp BF (1999) Fragebogen zu Selbstwirksamkeit, Optimismus und Pessimismus. Psychother Psychosom Med Psychol 49: 275–283.
22. Appels A (1997) Exhausted subjects, exhausted systems. Acta Physiol Scand Suppl 640: 153–154.
23. Hoerhold M, Klapp BF (1993) Testing the invariance and hierarchy of a multidimensional model of mood by means of repeated measurement with student and patient samples (Article in German). Z Med Psych 2: 27–35.
24. Schwarzer R, Lippke S, Luszczynska A (2011) Mechanisms of health behavior change in persons with chronic illness or disability: the Health Action Process Approach (HAPA). Rehabil Psychol 56: 161–170.
25. Hecheltjen KG, Mertesdorf F (1973) Development of a multidimensional mood questionnaire (Article in German). Gruppendynamik 40: 110–122.
26. Nowils V (1965) Research with the mood adjective checklist. In: Thompkins SS, editor. Affect, cognition and personality. New York: Springer.
27. Fliege H, Rose M, Arck P, Walter OB, Kocalevent RD, et al. (2005) The Perceived Stress Questionnaire (PSQ) reconsidered: validation and reference values from different clinical and healthy adult samples. Psychosom Med 67: 78–88.
28. Lowe B, Kroenke K, Herzog W, Grafe K (2004) Measuring depression outcome with a brief self-report instrument: sensitivity to change of the Patient Health Questionnaire (PHQ-9). J Affect Disord 81: 61–66.
29. American Psychiatric Association (2000) Diagnostic and statistical manual of mental disorders DSM-IV-TR. Washington, DC: American Psychiatric Press.
30. Kroenke K, Spitzer RL, Williams JB (2001) The PHQ-9: validity of a brief depression severity measure. J Gen Intern Med 16: 606–613.
31. Kroenke K, Spitzer RL, Williams JB, Lowe B (2010) The Patient Health Questionnaire Somatic, Anxiety, and Depressive Symptom Scales: a systematic review. Gen Hosp Psychiatry 32: 345–359.
32. Kroenke K, Spitzer RL, Williams JB, Monahan PO, Lowe B (2007) Anxiety disorders in primary care: prevalence, impairment, comorbidity, and detection. Ann Intern Med 146: 317–325.
33. Spitzer RL, Kroenke K, Williams JB, Lowe B (2006) A brief measure for assessing generalized anxiety disorder: the GAD-7. Arch Intern Med 166: 1092–1097.
34. Lowe B, Decker O, Muller S, Brahler E, Schellberg D, et al. (2008) Validation and standardization of the Generalized Anxiety Disorder Screener (GAD-7) in the general population. Med Care 46: 266–274.
35. Ellert U, Kurth BM (2013) [Health related quality of life in adults in Germany: results of the German Health Interview and Examination Survey for Adults (DEGS1)]. Bundesgesundheitsblatt Gesundheitsforschung Gesundheitsschutz 56: 643–649.
36. Ellert U, Lampert T, Ravens-Sieberer U (2005) [Measuring health-related quality of life with the SF-8. Normal sample of the German population]. Bundesgesundheitsblatt Gesundheitsforschung Gesundheitsschutz 48: 1330–1337.
37. Ware JE, Jr., Sherbourne CD (1992) The MOS 36-item short-form health survey (SF-36). I. Conceptual framework and item selection. Med Care 30: 473–483.
38. Stewart AL, Greenfield S, Hays RD, Wells K, Rogers WH, et al. (1989) Functional status and well-being of patients with chronic conditions. Results from the Medical Outcomes Study. JAMA 262: 907–913.
39. Byrnes B (2001) Structural equation modeling with AMOS. Mahwah: Erlbaum.
40. Tanaka JS (1993) Multifaceted conceptions of fit in structural equation models. In: Bollen KAL, J.S., editor. Testing structural equation models. Newbury Park: Sage.
41. Kocalevent RD, Hinz A, Brahler E, Klapp BF (2011) Determinants of fatigue and stress. BMC Res Notes 4: 238.
42. Altenhofer A, Schulz W, Schwab R, Eckert J (2007) Psychotherapy of adjustment disorders. Is psychotherapy if limited to 12 sessions sufficiently effective? Psychotherapeut 52: 24–34.
43. Gorschenek N, Schwab R, Eckert J (2008) [Psychotherapy of adjustment disorders]. Psychother Psychosom Med Psychol 58: 200–207.
44. Andreasen NC, Hoenk PR (1982) The predictive value of adjustment disorders: a follow-up study. Am J Psychiatry 139: 584–590.
45. Jones R, Yates WR, Zhou MH (2002) Readmission rates for adjustment disorders: comparison with other mood disorders. Journal of Affective Disorders 71: 199–203.
46. Catalina-Romero C, Pastrana-Jimenez JI, Tenas-Lopez MJ, Martinez-Munoz P, Ruiz-Moraga M, et al. (2012) Long-term sickness absence due to adjustment disorder. Occup Med (Lond) 62: 375–378.
47. Hapke U, Maske UE, Scheidt-Nave C, Bode L, Schlack R, et al. (2013) [Chronic stress among adults in Germany: Results of the German Health Interview and Examination Survey for Adults (DEGS1)]. Bundesgesundheitsblatt Gesundheitsforschung Gesundheitsschutz 56: 749–754.
48. Hogan JM, Carlson JG, Dua J (2002) Stressors and stress reactions among university personnel. Int J Stress Manage 9: 289–310.
49. Karakuş M (2013) Emotional intelligence and negative feelings: a gender specific moderated mediation model. Educ Stud 39: 68–82.
50. Kessler RC, McLeod JD (1984) Sex differences in vulnerability to undesirable life events. Am Sociol Rev 49: 620.
51. Sandanger I, Nygard JF, Sorensen T, Moum T (2004) Is women's mental health more susceptible than men's to the influence of surrounding stress? Soc Psychiatry Psychiatr Epidemiol 39: 177–184.
52. Slikovic A, Sersic DM (2011) Work Stress among University Teachers: Gender and Position Differences. Arhiv Za Higijenu Rada I Toksikologiju 62: 299–307.
53. Turner RJ, Avison WR (1989) Gender and depression: assessing exposure and vulnerability to life events in a chronically strained population. J Nerv Ment Dis 177: 443–455.
54. Day AL, Livingstone HA (2003) Gender differences in perceptions of stressors and utilization of social support among university students. Can J Beh Sci 35: 73–83.
55. de Smet P, Sans S, Dramaix M, Boulenguez C, de Backer G, et al. (2005) Gender and regional differences in perceived job stress across Europe. Eur J Public Health 15: 536–545.
56. Osorio LC, Cohen M, Escobar SE, Salkowski-Bartlett A, Compton RJ (2003) Selective attention to stressful distracters: effects of neuroticism and gender. Pers Indiv Differ 34: 831–844.

57. Eaton RJ, Bradley G (2008) The role of gender and negative affectivity in stressor appraisal and coping selection. Int J Stress Manage 15: 94–115.
58. Michael G, Anastasios S, Helen K, Catherine K, Christine K (2009) Gender differences in experiencing occupational stress: the role of age, education and marital status. Stress Health 25: 397–404.
59. World Health Organization (2003) Gender disparities in mental health. Geneva: World Health Organization.
60. Casey P, Pillay D, Wilson L, Maercker A, Rice A, et al. (2013) Pharmacological interventions for adjustment disorders in adults. Cochrane Db Syst Rev 2013.
61. Miller GE, Chen E, Zhou ES (2007) If it goes up, must it come down? Chronic stress and the hypothalamic-pituitary-adrenocortical axis in humans. Psychol Bull 133: 25–45.
62. Monteleone P, Nolfe G, Serritella C, Milano V, Di Cerbo A, et al. (2009) Hypoactivity of the hypothalamo-pituitary-adrenal axis in victims of mobbing: role of the subjects' temperament and chronicity of the work-related psychological distress. Psychother Psychosom 78: 381–383.
63. Hund B, Reuter K, Jacobi F, Siegert J, Wittchen H-U, et al. (2013) [Adaptation of the Composite International Diagnostic Interview (CIDI) for the assessment of comorbid mental disorders in oncology patients: the CIDI-O]. Psychother Psychosom Med Psychol [Epub ahead of print].
64. Stiles WB, Barkham M, Connell J, Mellor-Clark J (2008) Responsive regulation of treatment duration in routine practice in United Kingdom primary care settings: replication in a larger sample. J Consult Clin Psychol 76: 298–305.
